# A retrospective analysis of the relationship between rhinosinusitis and sinus lift dental implantation

**DOI:** 10.1186/1746-160X-10-53

**Published:** 2014-12-15

**Authors:** Gurkan Kayabasoglu, Alpen Nacar, Aytug Altundag, Melih Cayonu, Mehmet Muhtarogullari, Cemal Cingi

**Affiliations:** Otolaryngology Head and Neck Surgery Department, Sakarya University Medical School, Adnan Menderes Cad No.145, Adapazarı Sakarya, Istanbul, Turkey; Department of Otorhinolaryngology, Faculty of Medicine, Sakarya University, Sakarya, Turkey; Otolaryngology Department, Istanbul Surgery Hospital, Istanbul, Turkey; Otolaryngology Department, Amasya University Research and Training Hospital, Amasya, Turkey; Dentistry Department, Hacettepe University, Sihhiye Kampusu, 06100 Turkey; Otolaryngology Head and Neck Surgery Department, Osmangazi University, Eskisehir, 26100 Turkey

**Keywords:** Dental implant, Maxillary sinusitis, Rhinitis, Complications, Sinus lift

## Abstract

**Introduction:**

Dental implants have been associated with the occurrence of postoperative rhinosinusitis. In some patients, preoperative sinus lifting must be performed to increase the chances of successful implant placement. This retrospective study examines the relationship of dental implants after sinus lifting with the occurrence of postoperative rhinosinusitis.

**Methods:**

A total of 268 dental implants were inserted in 94 patients (62 Males, 32 Females) between 2011–2013. The ages ranged from 29–71 (in males) and 33–64 (in females). Additionally, bilateral sinus lifing was performed in 51 patients, and unilateral sinus lifting was performed in 43 of the patients. Patients were evaluated for sinus pathology for a period of 5–47 months postoperatively using a satisfaction questionnaire, conventional radiographic examination, and nasal endoscopic examination.

**Results:**

Four of the patients (4.2%) had a complication of postoperative sinusitis and were treated medically. In one patient, the implant was unsuccessful (even after treatment) and was removed. None of the patients needed surgery due to the sinusitis or any associated complications.

**Conclusion:**

The risk for postoperative rhinosinusitis was found to be higher in patients who suffer from chronic sinusitis and in cases in which a large amount of graft was utilized for sinus lifting. These predisposing factors need to be considered when evaluating patients for dental implants and sinus lift procedures.

**Electronic supplementary material:**

The online version of this article (doi:10.1186/1746-160X-10-53) contains supplementary material, which is available to authorized users.

## Introduction

**Figure 1:**
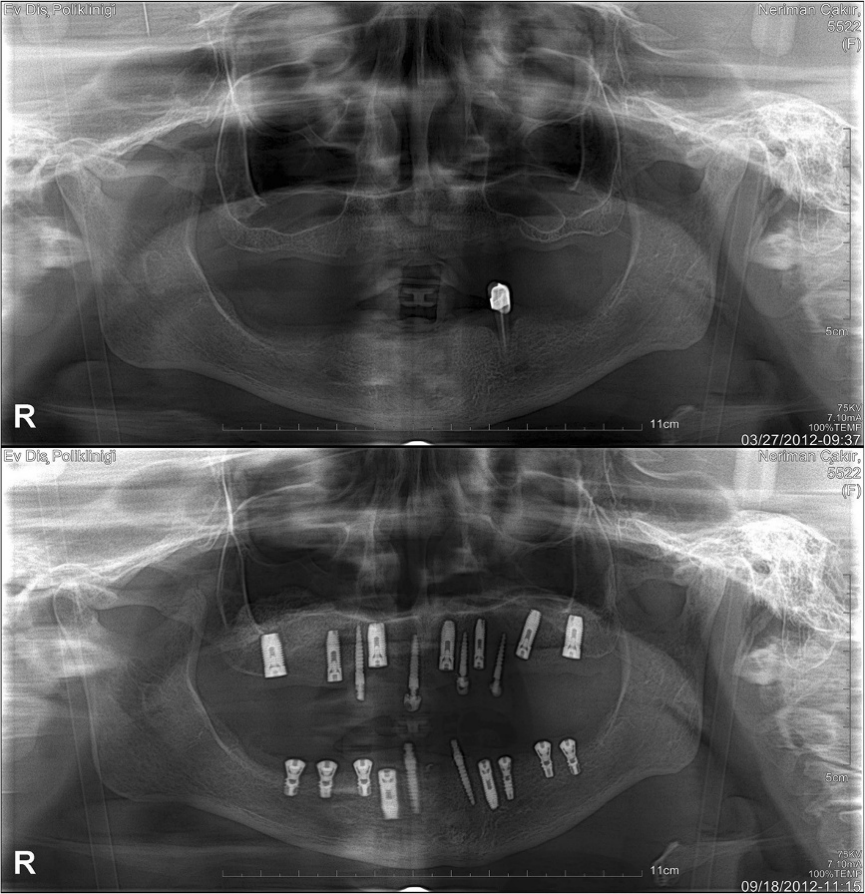
**An edentulous patient’s comparative panoramic x-rays before and after dental implant treatment.**

The introduction of endoseous dental implants as an option for partially and fully edentulous patients has revolutionized dental treatment. Dental implants are commonly composed of a titanium material screw and crown that are surgically placed in the jawbone. The implant becomes osseointegrated within a few months, allowing it to withstand chewing and biting forces, analogous to natural tooth function. Common indications for undergoing a dental implant procedure include: replacement of a missing tooth/teeth, replacement of multiple teeth with a secured bridge implant and increased support of removable partial/full dentures [1] (Figure 1).

The posterior edentulous maxilla is often seen as a challenge for the oral surgeon as alveolar ridge reabsorption and maxillary sinus pneumatization decreases the bone available for implant stabilization. As such, edentulous alveolar ridges are considered unfavorable for implant placement [2–4]. In cases where there is insufficient bone to provide support for dental implants, bone grafting may be considered. Dentists often perform sinus mucosal lifting procedures to increase the safety factor of bone grafting [5]. Autogenous bone grafting to augment the maxillary sinus floor is a generally accepted pre-implant procedure that facilitates the successful placement of endosseous implants in the correct prosthetic position [6, 7]. In these cases, complications related to sinusitis can occur during the grafting of bone, during the sinus lift, or after the completion of the sinus lift [2].

**Figure 2:**
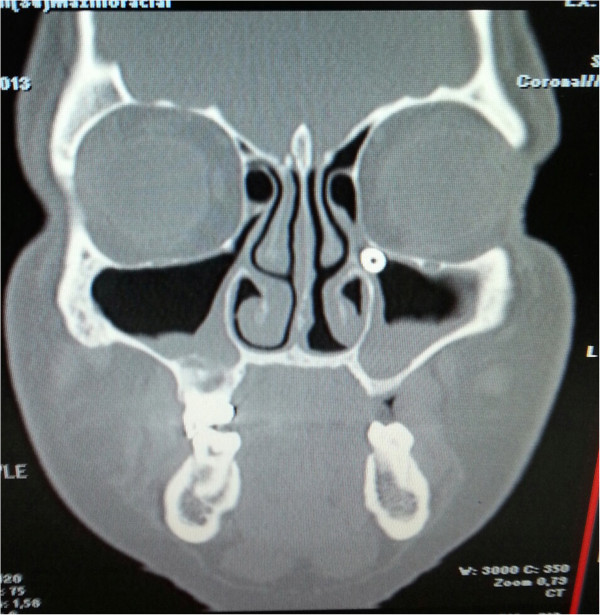
**A rare complication of dental implantation, sinusitis due to a migrated dental implant to the maxillary sinus ostium.**

Many other complications of dental implants have been documented in literature: bleeding, inflammation, dental implant rejection, dental implant overload, failure of dental implant, bone loss, implant migration to the sinus or nasal cavity, incision line opening, infection, fractures, and fat embolism in the mandible [4–7] (Figure 2).

In contrast to reports of failure of osseointegration, there are very few descriptions of implant rejection since a majority of the implants being used today are made of titanium, a biocompatible material. Additionally, human corticocancellous mineralized allograft bone has been shown as a suitable graft material choice in maxillary sinus augmentation [5].

As the maxilla is composed of low density cortical bone and short alveolar ridges, there exists the possibility of dental implant failure with other complications such as: maxillary sinusitis, oroantral fistula, and displacement of the dental implant to the maxillary sinus.

Rhinosinusitis is one of the most common diseases in Western societies, causing significant morbidity and resulting in great financial cost to the patient. Although multiple theories have been proposed regarding the underlying pathogenesis (including: allergy, bacterial or fungal infection, genetic predisposition and structural anomalies) at present, the majority of cases are still considered idiopathic [8–12]. In the long list of etiologies, one factor is dental implantation and related post-implant complications [13].

Rhinosinusitis, defined as inflammation of the nose and paranasal sinuses, most often presents as patients seek medical attention to relieve nasal blockage and discharge. Facial pain/pressure and hyposmia (decrease in the sense of smell) are considered minor symptoms according to the EPOS of 2012. Patients may also suffer from headache, dental pain, halitosis, fatigue, cough, and ear pain during sinusitis [11–15]. Specifically, the reported minor symptoms are common findings with sinusitis occurring as a result of dental infections, therefore it is important to closely and carefully monitor patients in their post-operative follow-up [11, 12].

This retrospective study aims to investigate the relationship of dental implants after sinus lifting with the occurrence of postoperative rhinosinusitis.

## Methods

In this retrospective case control study, conducted in full accordance with the World Medical Association Declaration of Helsinki and collection of an informed consent from the patients, after an institutional ethical board approval, the records of 94 consecutive patients who received a dental implant between January 2011 and January 2013 at Ev Private Dentistry Clinic were reviewed. A total of 268 implants were placed in these patients, and all had a minimum sinus floor thickness of 5 mm. After local and regional anesthesia administration, all patients underwent a lateral window approach. The sinus membrane was carefully elevated from the sinus floor and medial sinus wall. Human corticocancellous mineralized allograft bone was used as graft material. The same graft materials and implantation techniques were used on all patients. Submerged implants were placed with a drill speed of 750 rpm immediately following the sinus lifting, and prosthetic loading was performed 6 months following the implant placement. Collagen barrier membranes were utilized in cases of mucosal perforation due to manipulation of the area, but otherwise none were placed over the lateral window. The implant did not contact the sinus membrane in any of the patients.

Patients were evaluated for sinus pathology for a period of 5–47 months after bone transplantation and implant insertion using a SNOT-22 questionnaire postoperatively, both pre-operative and post-operative panoramic radiological imaging was employed to monitor the progress of all patients. None of the patients underwent a preoperative CT scan for the purposes of diagnosing their sinus pathology. All patients were questioned for complaints and symptoms of sinusitis preoperatively, and any positive findings were assessed by an otolaryngology consultation. In patients with a diagnosis of sinusitis, an otolaryngologist then performed a full work-up and examination (with nasal endoscopy and CT Scan) and treated the patient accordingly.

Patients were included in the study according to the following criteria: area of missing teeth, a minimum sinus floor thickness of 5 mm, asymptomatic sinus disease, and open airflow. Patients who were either noncompliant with appointments and/or follow-up procedures or had acute sinusitis, were excluded from the study.

## Results

**Figure 3:**
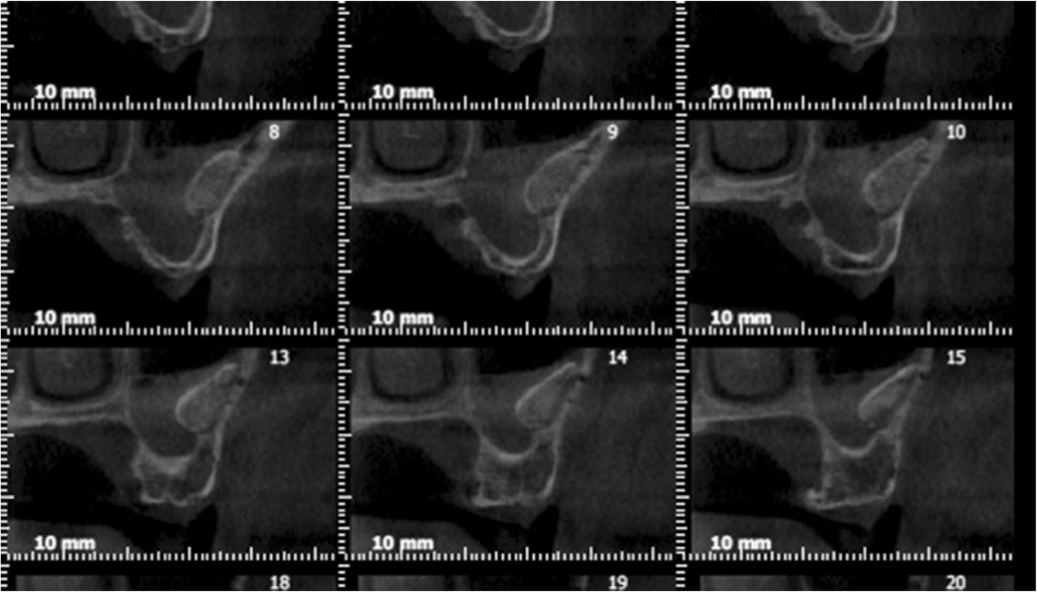
**Maxillary sinusitis after sinus lifting and bone graft.**

A total of 268 dental implants were inserted in 94 patients (62 Males, 32 Females) between 2011–2013. The ages ranged from 29–71 (in males) and 33–64 (in females.). 145 sinus lift procedures (bilateral in 51 patients, unilateral in 43 patients) were performed during the implantation. Postoperative unilateral maxillary sinusitis was detected in 4 of 94 patients; these 4 patients had undergone bilateral sinus lifting. (Figure 3) Of these patients, 3 had reported chronic sinusitis in their history, and 1 required an unusually high volume of graft material due to increased maxillary reabsorption. Two of the 4 patients also had ipsilateral ethmoid sinusitis (Additional file 1), 3 of the 4 patients had suffered from purulent exudative leakage from an intraoral fistula, and 1 had symptoms of mild acute sinusitis. For the patients with an intraoral fistula, infected graft materials were aspirated from sinus cavity and they were placed on a 10-day course of clindamycin. 2 of the 4 patients exhibited total recovery. 1 patient lost an implant due to a lack of response to the treatment, and the other was given an additional 10-day course of amoxicillin-clavulanic acid and exhibited full recovery. No further surgical intervention was required in any of the patients.

## Discussion

Sinusitis can occur as a result of contamination of the maxillary sinus with oral flora in aseptic surgical conditions [16]. Although iatrogenic small sinus membrane perforations during surgery does not seem to be related to the development of postoperative sinusitis in healthy patients, large perforations of the maxillary sinus membrane have a higher likelihood of resulting in a discharge of bony fragments into the maxillary sinus and leading to maxillary sinusitis. Other causes are: ostium obstruction due to postoperative swelling of the maxillary mucosa, blockage of air flow due to diminished intrasinus volume, impaired mucosal activity in the maxillary sinus due to mucosal lacerations, and implant exposure and extensions [17–19].

In their report of 156 dental implant patients with no intraoperative surgical complications, Timmenga et al. showed that small perforations of the sinus membrane (less than 2 mm in diameter) with exposure into the maxillary antrum would often heal spontaneously with normal blood clot formation and routine mucosal healing [2]. As well, in their patients the mucosal lining of the maxillary sinus was not perforated. During postoperative follow-up, 7 patients (4%) developed transient (subacute) sinusitis, and 2 patients (2%) developed chronic purulent maxillary sinusitis.

As surgical procedures themselves can be a possible cause of sinusitis, all patients were operated on under strict adherence to antisepsis guidelines. In our study, the lateral window approach along with submerged implants were used as they are believed to be safer. We used 1–2 mm of allograft for the procedure, since the reabsorption rates can be higher with larger granules, leading to an increased risk of contact between the implant and sinus membrane. Sinus membrane perforation was detected in 8 patients, and a collagen membrane barrier was used to cover the perforation. None of the 8 patients with perforation were part of the group who developed sinusitis.

There have also been studies on the effects of dental implants exposed to the sinus cavity in relation to sinus complications. Raghoebar et al. reported that implants that have undergone extension into the nasal cavity can give rise to rhinosinusitis, and the most likely explanation for this complication is that altered nasal airflow could induce irritation of the nasal mucosa [8, 20]. Figure 2 shows a patient (from a different clinic) who underwent implant placement without sinus lifting and developed subsequent implant-related rhinosiunistis, and is an example of a finding in accordance with the study by Roghoebar et al..

In their study, Sbordone et al. found that the amount of remodeling of the implanted graft depends on the nature of the inlay graft (bovine bone material or autogenous bone from the chin and the iliac crest) with regard to apical measurement, and on the type of procedure of implant insertion (simultaneous or delayed) with regard to cervical measurement. As there was no reported difference in the implant outcome between simultaneous and delayed insertion, we chose to insert the allografts simultaneously. None of the patients in our study experienced extrusion of the implant and no such etiology was found in the patients who developed sinusitis [21–24]. Furthermore, literature shows that not every case of implant extrusion will lead to sinusitis.

Jung et al. reviewed 9 retrospective cases in which 23 implants were placed in the maxillary sinus in such a manner as to allow penetration of the sinus floor by more than 4 mm (mean 5 mm, range 4–7 mm) without lifting the sinus mucous membranes. None of the patients experienced sinusitis, and the study concluded that implant exposure to the maxillary sinus cavity can induce sinus mucous thickening around the implants. They did note, however, that studies involving longer time intervals might be necessary to determine whether the mucosal thickening can become a source of sinusitis [20, 25].

In an animal experimental study, Jung et al. showed that in cases that implants penetrated the mucosa of the sinus floor by more than 4 mm, the portions of the implants extending into the sinus cavity were not fully covered with a newly formed sinus membrane. Therefore, one might expect that implants protruding into the sinus cavity could act as a foreign body and become a source of inflammation and sinusitis. In addition, nasal clearance could be disturbed by implant blockage of the mucociliary pathway, again giving rise to inflammation [20, 26]. None of the patients in the study had a direct penetration of the implant into the sinus cavity, and although some graft reabsorption was seen in the 6-month postoperative radiographs, in no case was the implant in direct contact with the sinus mucosa.

Implant migration is the most serious form of the implant exposition in sinus cavity, and in these cases, patients suffer from severe sinusitis symptoms surgical intervention often performed after completion of medical therapy [27, 28].

One of the limitations of the study is the possibility that patients had a pre-existing localized sinus pathology, which led to postoperative sinusitis. Although this limitation is difficult to avoid due to retrospective nature of the study, those patients who either had complaints of acute sinusitis or questionable results on their panorex film were either seen and cleared by an otolaryngologist or excluded from the study.

## Conclusion

As the number of surgical procedures to place implants in the posterior maxilla is rapidly expanding, such complications will probably increase in the future. Studies show that patients who have a history of sinusitis are at a higher risk for developing post-operative sinusitis following a dental implant. Because of this, all patients should be screened for history of sinusitis, and in those who do have a history, a consultation from an Otolaryngologist should be considered as a way of decreasing the risk of complications and increasing the success of the procedure. This will ensure that the patient’s health will not be at risk during the implant or the follow up.

## Electronic supplementary material

Additional file 1: Figure S1: An edentulous patient’s comparative panoramic x-rays before and after dental implant treatment. (JPEG 109 KB)
