# Supplementary figures and images for: A retrospective analysis of the relationship between rhinosinusitis and sinus lift dental implantation
Source: Head Face Med. 2014 Dec 15;10:53. doi: 10.1186/1746-160X-10-53 (PMC4414305; doi:10.1186/1746-160X-10-53)

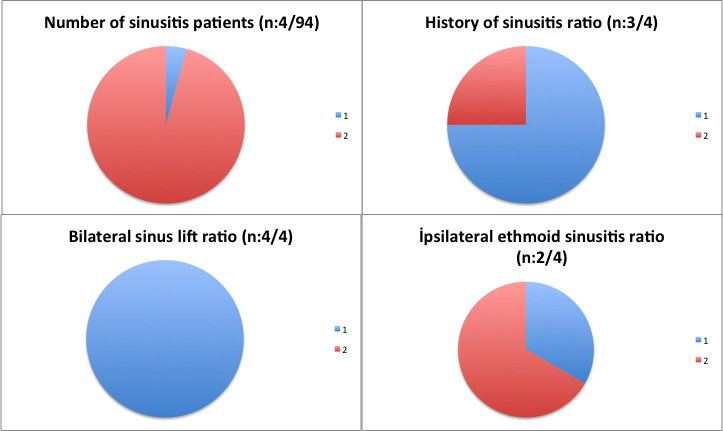

Supplement: Supplementary file 1 — Additional file 1: Figure S1: An edentulous patient’s comparative panoramic x-rays before and after dental implant treatment. (JPEG 109 KB) [file 13005_2014_388_MOESM1_ESM.jpeg]
